# DNA damage repair defects as a new class of endocrine treatment resistance driver

**DOI:** 10.18632/oncotarget.26363

**Published:** 2018-11-20

**Authors:** Meenakshi Anurag, Matthew J. Ellis, Svasti Haricharan

**Affiliations:** Svasti Haricharan: Department of Tumor Microenvironment and Cancer Immunology, Sanford Burnham Prebys Medical Discovery Institute, La Jolla, CA, USA

**Keywords:** endocrine treatment resistance, DNA repair

## Beyond homologous recombination defects in breast cancer

Cancer cells constantly balance the cost of incurred DNA damage against the benefit of uninhibited proliferation. In the past decade, translational advances have enhanced our understanding of diverse cellular processes associated with tumor genome integrity that impact this balance, and therefore, can be leveraged as therapeutic opportunities. In breast cancer, the emphasis of investigations into DNA damage pathways and tumor outcomes has been germline variants that affect tumor incidence, as exemplified by *BRCA1/2* and to a lesser extent, *PALB2*, *ATM*, *CHEK2*, *RAD51*, *TP53* among others [1]. Among these components, BRCA1 and BRCA2, belonging to the homologous-recombination pathway have been most widely studied in ovarian and breast cancer. In 2003, a seminal report examined lifetime risk of breast and ovarian cancer in women with BRCA1/2 germline mutations [2], propelling investigation into the role of BRCA1/2 in breast cancer in the context of tumor incidence, tumor biology and reproductive events. Such studies established the prevalence of “BRCAness” in estrogen-receptor negative breast cancers and lead to the concept of creating synthetic lethality in BRCA2-deficient cells by treating them with PARP-inhibitors [3]. However, the role of somatic defects in other DNA repair pathways in breast cancer biology and clinical outcome remained understudied.

## New class of drivers of endocrine treatment resistance: Single strand break repair pathways

**Figure 1:**
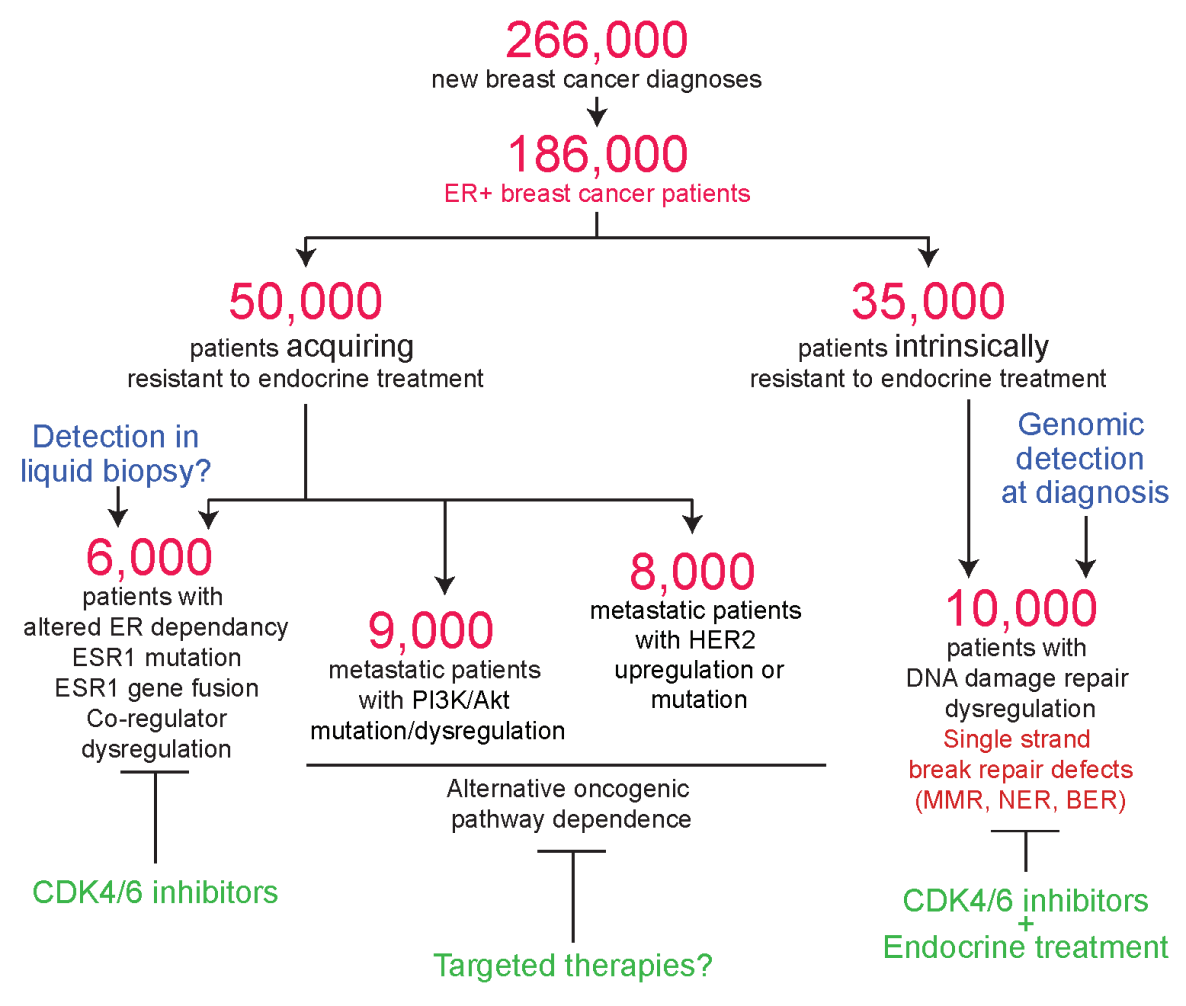
Flow chart depicting the number of predicted endocrine treatment resistant patients in the USA, and attributing known mechanisms of resistance based on estimated frequencies reported in the literature Where evidence exists, putative diagnosis and therapeutic strategies to target each mechanism is included.

About three quarters of breast cancers are estrogen receptor positive (ER+), i.e they express estrogen receptor at a level detectable by immunohistochemistry. Although these cancers tend to be less immediately aggressive than other subtypes, between 40 and 50% of ER+ patient tumors demonstrate resistance to standard-of-care endocrine therapy with many patients relapsing 5 or more years after diagnosis (Figure 1). Resistance can be broadly classified as either acquired or intrinsic, meaning resistance arises after treatment with endocrine interventions or that resistance is innate within the tumor rendering it instantly and preemptively resistant to endocrine interventions. Of these two mechanisms of resistance, acquired resistance is the best studied.

Mutations in *ESR1* as well as activation of growth factor pathways, e.g. HER2, have been well established as drivers of acquired resistance in preclinical studies and in clinical data from patient tumors [4]. More recently, *ESR1* gene fusions were also identified as drivers of acquired resistance in patients with metastatic ER+ breast cancer [5, 6]. These combined insights into the underlying biology of acquired endocrine treatment resistance in as many as 40% of resistant patients has resulted in potentially more effective anti-estrogens that are currently being tested in clinical trials.

On the other hand, drivers of intrinsic resistance have been understudied, with the notable exception of *HER2* amplification [7], the discovery of which reclassified ER+ breast cancer and significantly improved therapeutic options. Two recent studies identified defects in DNA damage repair genes belonging to single strand break repair pathways, primarily mismatch and excision repair, as an entirely new causal mechanism observed in ∼1/3rd of intrinsically endocrine treatment resistant ER+ breast cancer patients (Figure 1) [8, 9]. These results suggest that distinct pathways may be dysregulated in patient tumors that are intrinsically resistant to endocrine treatment, and open new avenues for improvement of diagnostic and therapeutic clinical space.

## Promise for new therapeutic and predictive avenues –prediction of sensitivity to CDK4/6 inhibitors

Preclinical causal and mechanistic investigation into the role of single strand break repair pathways in endocrine treatment resistance suggested a common mechanism of dysregulated G1-S transition by which mutation or downregulation of select mismatch repair and excision repair genes lead to endocrine treatment-resistance [8, 9]. Loss of any of the specific mismatch, nucleotide excision or base excision repair components identified leads to unchecked activation of CDK4 even in the presence of endocrine treatment, rendering these tumors resistant to endocrine treatment but sensitive to CDK4/6 inhibitors in combination with endocrine treatment [8, 9]. This discovery presents the use of CDK4/6 inhibitors (e.g. palbociclib, abemaciclib) as front-line therapy in ER+ breast cancer patients, thereby increasing chances of preventing resistance and metastasis. In a recent study, selective CDK4/6 inhibitors where shown not only to induce tumor cell cycle arrest, but also promote anti-tumor immunity [10], hence providing rationale for new combination regimens comprising CDK4/6 inhibitors and immunotherapies as anti-cancer treatment. High mutation load consequent of these endocrine therapy resistance-inducing single stand break repair defects should further contribute to the immunogenicity of these tumors. These discoveries also lay the foundation for new diagnostic assays that can stratify patients early on in the timeline of their disease as likely to respond, or not, to endocrine treatment and CDK4/6 inhibitor treatment, a potential breakthrough in effective clinical management of breast cancer.

Overall, advances in translational research have identified potential causes of acquired endocrine treatment resistance in 30-40% of breast cancer patients resulting in an escalation of clinical investigations testing (Figure 1)targeted therapies that will undoubtedly present clinicians with more options when treating their patients. Recent discoveries of a role for DNA repair defects will likely similarly impact clinical treatment for patients with ER+ breast tumors that are intrinsically resistant to endocrine treatment. Continuing studies and new insights into the biology underlying this condition provide promise of truly effective personalized medicine for this subset of patients.
